# Respiratory Symptoms in Relation to Living near a Crude Oil First Treatment Plant in Italy: A Cross-Sectional Study

**DOI:** 10.3390/ijerph15122636

**Published:** 2018-11-25

**Authors:** Elisa Bustaffa, Alessio Coi, Fabrizio Minichilli, Michele Santoro, Renato Prediletto, Simonetta Monti, Ivana Pavlickova, Fabrizio Bianchi

**Affiliations:** 1 National Research Council, Institute of Clinical Physiology, Unit of Environmental Epidemiology and Disease Registries, Via Moruzzi 1 56100 Pisa, Italy; elisa.bustaffa@ifc.cnr.it (E.B.); alessio.coi@ifc.cnr.it (A.C.); fabrizio.minichilli@ifc.cnr.it (F.M.); michele.santoro@ifc.cnr.it (M.S.); 2 Gabriele Monasterio Foundation National Research Council-Tuscany Region, Institute of Clinical Physiology, Via Moruzzi 1 56100 Pisa, Italy; predile@ifc.cnr.it (R.P.); monti@ifc.cnr.it (S.M.); pavlickova@ifc.cnr.it (I.P.)

**Keywords:** crude oil, air pollution, respiratory symptoms, cross-sectional, questionnaire

## Abstract

Exposure to air pollution has been shown to increase the risk of developing chronic respiratory diseases. The largest crude oil first treatment plant in Italy emits harmful polluting substances. This cross-sectional study assesses the occurrence of respiratory symptoms in a sample of the adult population living near the plant. A proximal and a reference area were defined in order to recruit 200 subjects. Each subject performed a spirometry test and completed a questionnaire. Associations between the distance from the plant and selected respiratory outcomes were assessed (distance-based approach). The prevalence of outcomes between a proximal and a reference area (area-based approach) were also compared. Odds ratios were adjusted for potential confounders. Living near the plant was associated with a higher prevalence of respiratory symptoms, with significant associations for severe dyspnoea equivalent to the halving of risk as the distance of residence from the plant increased by 1 km (adjusted odds ratio (OR) 0.48, confidence interval at the 95% probability level (95% CI): 0.25–0.92). Several signals emerged for respiratory allergic symptoms. The area-based approach generally confirmed the results obtained with the distance-based approach. This is the first study to be carried out on a crude oil first treatment plant aimed at assessing the association with the occurrence of respiratory symptoms. These findings contribute to the evidence supporting the need for a space–time surveillance system in the investigated area.

## 1. Introduction

The largest crude oil first treatment plant in Italy is in Val d’Agri, in Basilicata. It is located in an anthropized area at the bottom of a valley, between the municipalities of Viggiano and Grumento Nova, and began operating in 2001. It has a surface of 180,000 m^2^ and a nominal treatment capacity of 104,000 barrels of oil per day and 4,660,000 standard cubic meters of gas per day. Its emissions derive from stationary combustion, gas flaring and gas venting, with fugitive emissions from oil storage tanks, valves, vents, etc. The plant in Val d’Agri is not in fact a standard oil refinery, but only carries out pre-treatment. In fact, the only process these two kinds of plants have in common is the initial treatment; that is, the desulfurization process. The extracted oil is either directly conveyed into the refinery through pipelines or passes beforehand into a first treatment plant, or into an oil collection center, before being transferred to the refinery. The first treatment process is based on the three-phase separation of crude oil from which gases, treated crude oil and water are extracted. Particularly, in oil refineries, the operations that can lead to pollutant emissions are the handling of products, the filling and emptying of tanks and the treatment of industrial waste water [1]. These operations are also carried out in crude oil first treatment plants but, in the oil refineries, distillation products are also stored and the waste water also comes from industrial processes. The substantial differences in the processes have relevant consequences in term of their pollutant emissions and health effects.

The plant emits nitrogen oxides (NOx), sulfur dioxide (SO_2_), carbon monoxide (CO), particulate matter, volatile organic compounds (VOCs) and non-methane hydrocarbons (NMHCs), a subgroup of VOCs [2]. These pollutants are produced during various phases, particularly during combustion phenomena such as torching. In these cases, after the separation of the solid and liquid species, the gaseous compounds present in the plants flow to the blowdown system (torches), where they are burnt on the top using atmospheric oxygen [3]. Currently, the emissions and the concentrations in the atmosphere of all the VOCs are not regulated by law either at the European or national level.

In the study area, there has been a lack of continuous environmental monitoring and epidemiological surveillance of the resident populations aimed at assessing the health impacts attributable to the plant’s activities. Therefore, between 2014 and 2017, the “Health Impact Assessment in the Municipalities of Viggiano and Grumento Nova in Val d’Agri” project was carried out [4]. As part of this project, a cross-sectional study on respiratory function was carried out on a sample of subjects residing in the municipalities of Viggiano and Grumento Nova who were exposed to atmospheric pollution produced by the crude oil first treatment plant.

The aim was to investigate respiratory symptomatology in an area with a crude oil first treatment plant. In fact, epidemiological studies on populations exposed for long periods to atmospheric pollution have showed an increased risk of reduced lung function [5,6,7,8]. Short-term exposure to elevated concentrations of atmospheric pollution are known to be associated with an increased frequency of irritative inflammatory reactions in the upper airways and with respiratory symptoms such as cough or wheezing [9,10]. Also, epidemiological studies on populations residing in the proximity of an industrial/petrochemical complex showed an increase of acute irritative symptoms of the respiratory tract and eye, and asthma [11,12], an excess risk of respiratory diseases [13], acute respiratory problems [14], hospitalization for asthma, acute respiratory infection and chronic obstructive pulmonary disease (COPD) [15]. Particularly, a residential cohort study conducted on populations of Viggiano and Grumento Nova showed excesses of hospitalization for respiratory diseases [16].

The World Health Organization (WHO) recently estimated that 35% of respiratory diseases could be attributable to the environment and proposed interventions in industrial, commercial, transport, housing/community sectors to reduce population exposures to air pollution [17].

The aim of this cross-sectional study is to assess whether symptoms and respiratory health were associated with distance of residence from a crude oil first treatment plant. To the best of our knowledge, this is the first study to investigate the effects on the respiratory system in populations residing near a crude oil first treatment plant.

## 2. Materials and Methods

The survey included spirometry tests and the concurrent administration of a questionnaire aimed at investigating the symptomatology of the respiratory system (presence of cough and sputum, dyspnoea, respiratory allergic symptomatology, chronic bronchitis, and asthma). The questionnaire enabled us to investigate the conditions and lifestyle of the subjects involved in the study in order to improve the characterization of exposure and to control for possible confounding factors during the analysis.

### 2.1. Sample Selection

As part of the study, 200 subjects were recruited who resided in Viggiano and Grumento Nova within the area defined by the total of the two administrative areas of the municipalities. In the valley, the prevailing wind direction transports pollutants from south-west and north-west sectors towards the east and north-east, and the complexity of the orography drives the distribution of pollutants at the ground. A proximal area (PA) and a reference area (RA) were thus defined within the study area by an ellipse of major and minor axes of 12 and 4 km, respectively, centered on the plant. The PA and RA boundaries do not match with the municipal ones. The definition of these areas was used to perform the sample selection, recruiting 100 subjects residing in the PA and 100 subjects residing in the RA (see Supplementary Materials—Method for the sample extraction). In particular, a random selection stratified by gender, age group (18–75 years), and municipality was performed. The residence addresses of all the subjects were obtained from municipal registries. Before the extraction, all the residents in the two municipalities were georeferenced and classified as resident in the PA or in the EA. Geocoding of addresses was performed using the geographic information system software, ArcGIS 9.2 (ESRI, Redlands, CA, USA).

Subjects were directly contacted and recruited by the nursing staff of the Pneumology Complex Operating Unit of the Villa d’Agri Hospital Presidium. During recruitment, whenever a subject refused to participate in the survey, a matching substitute (gender, age, municipality and area (PA/RA) of residence) was found.

### 2.2. Questionnaire

A questionnaire was administered to the 200 recruited subjects, structured according to the methods and tools used in previously performed surveys [18,19]. The questionnaire covered personal information, cigarette smoking habits, presence and severity of respiratory symptoms, past respiratory history and general medical history, environmental and socio-economic conditions, diet, type of employment (including unemployed), and exposure to chemical agents.

Questionnaires were administered by the nursing staff who had been specifically trained for the administration of questionnaires. The questionnaire was digitized using SurveyGizmo software (SurveyGizmo, Boulder, CO, USA).

### 2.3. Spirometry Test

Respiratory function tests were performed by the nursing staff in accordance with the European Respiratory Society and the American Thoracic Society standards [20] in order to assess the presence of a chronic airflow obstruction. The questionnaire and spirometry were performed on the same day by the same nursing staff. Lung function studies included the measurement of forced vital capacity (FVC) and forced expiratory volume in one second (FEV_1_). Briefly, the subjects were connected to a spirometer by a mouthpiece in sitting position and asked to take a maximal inspiration; they were then requested to breathe out as forcefully as they could. FVC is the air volume (in liters) blown out at the end of the manouver; FEV_1_ is the air volume exhaled in the first second of the manouver. The ratio of the two measurements (FEV_1_/FVC %) was calculated. At least three spirometric measurements were obtained, and the highest values were chosen. A Vmax Sensor Medics spirometer was used, with flows and volumes calibrated according to the guidelines. Spirometric values were evaluated by comparison with reference values based on age, sex and height and expressed as percentages of predicted values (with the exception of FEV_1_/FVC %) [21]. In subjects with airflow limitation, defined as FEV_1_/FVC < 70%, a bronchodilator test was performed by administering a β2-short acting bronchodilator (salbutamol 400 mcg), then repeating an FVC manouver after 15 minutes delay; chronic airflow obstruction was diagnosed for a fixed post-bronchodilator FEV_1_/FVC < 70% [22]. The severity of airflow obstruction was expressed according to the values of FEV_1_% pred, as follows: mild (FEV_1_ ≥ 80% pred), moderate (50% ≤ FEV_1_ < 80% pred), severe (30% ≤ FEV_1_ < 50% pred), very severe (FEV_1_ < 30% pred) [23]. Spirometric data were then digitized.

### 2.4. Respiratory Outcomes

The outcomes selected for the study were derived both from the respiratory function test (presence of chronic airflow obstruction; FVC% pred) and the respiratory symptoms obtained from the questionnaire (presence of cough and sputum, alone or in association, outside common colds, for some periods of the year and possibly for at least two years; presence of dyspnoea; history of chronic bronchitis; history of bronchial asthma or asthmatic bronchitis; allergic respiratory symptoms in association (or not) with symptoms on eyes (pruritus, redness and burning)).

### 2.5. Statistical Analysis

The association between the distance from the centroid of the plant and the investigated outcome was assessed using the distance included in a multivariate model as a continuous variable (distance-based approach). The odds ratio was thus interpreted as a change in the odds associated with a 1 km increment from the residence to the plant; an OR < 1 represents a potential prevalence reduction with each kilometer away from the plant.

Differences in the occurrence of the investigated outcomes between the group of residents in the proximal area and the group of residents in the reference area (defined as reported in Section 2.1.), were also assessed (area-based approach). Descriptive analyses were performed in order to assess differences between the two areas in terms of their outcomes and potential risk factors.

Multivariate analyses using the logistic regression model were performed both for the distance- and the area-based approach for each of the outcomes considered, using the likelihood ratio test to fit the model. Well known risk factors for respiratory symptoms and respiratory function were included a priori in the multivariate models: gender; age; body mass index (BMI) defined as kg/height2; smoking (expressed in packs per year = (number of cigarettes per day*number of years of smoking)/20); cardiovascular comorbidity (acute myocardial infarction or ischemic heart disease; stroke or transient ischemic attack; obliterative arterial disease of the lower limbs; deep vein thrombosis; embolism); exposure to chemical–physical agents (dust, gases and fumes); and distance (buffer of 500 m) from the main road in the valley. Other variables found to be associated (*p* < 0.05) with both the distance from the plant and the outcome were added to the model. Covariates examined for entry into regression models included respiratory symptoms (bronchitis, pneumonia at least once or twice a year during cold seasons, pertussis or croup/pseudo croup) in pediatric age (up to the age of 12); metabolic comorbidities (jaundice, liver disorders or colic, liver failure, hepatitis; high cholesterol and/or triglycerides in the blood; diabetes, excluding gestational); family history of respiratory diseases (in mother, father and, eventually, brother/sister) (emphysema, chronic bronchitis, bronchiectasis, bronchial asthma, tuberculous, lymph-glycol, parenchymal or pleural processes, lung tumors); homes with wood heating; being employed/unemployed; and being employed (current)/unemployed in industries.

For the analyses of bronchial asthma and allergic symptomatology (also in association with eye symptomatology), the same analysis was conducted including the following pre-defined covariates: gender, age, BMI (for bronchial asthma only), smoking (packs per year = (number of cigarettes per day*number of year of smoking)/20), distance from the main road, and exposure to chemical–physical agents (dust, gases and fumes).

Descriptive analyses were performed and presented using the chi-square test. The results are reported as frequency and percentage (categorical variables) or mean ± standard deviation (SD) (continuous variables). A value of *p* < 0.05 was used as a threshold to define a statistically significant result. The results of the multivariate analysis were presented and discussed for outcomes with at least 10 subjects (5% of the study sample). The odds ratio (OR) was calculated with a confidence interval at the 95% probability level (95% CI) and the exact value of the type I error probability (p).

All the analyses were performed using the STATA software version 13.1 (StataCorp LP, College Station, TX, USA). 

The study was carried out in accordance with the Declaration of Helsinki. The study was authorized by the Local Health Unit of Potenza (ASP) (Deliberation of the ASP General Director number 2015/00678 of the 2015/10/16), which is the holder of the health data. All the study activities were described in a written agreement between the ASP and the Institute of Clinical Physiology of the Italian National Research Council (IFC-CNR). The agreement provided for the anonymization of personal data, which were only permitted to be managed by the institutions that had signed the agreement. All subjects gave their informed consent for inclusion before participating in the survey. No personal identifiers were sent to the research staff, all addresses were geocoded and the personal data were analysed anonymously, as required by the convention.

## 3. Results

A total of 3641 subjects, aged 18–74, were extracted from the registries of the two municipalities (on 31 December 2014). We contacted 263 subjects in order to meet our target of 200 responders. A total of 77% of contacted subjects participated in the study.

The spirometric test and the administration of the questionnaire were performed on the final sample of 200 subjects. Nine subjects were excluded from the sample for the statistical analyses after a quality control check for the following reasons:

- Eight subjects (6 from Grumento Nova and 2 from Viggiano) were only resident for short periods, thus not adhering to the continuity criterion;
- One subject (from Viggiano) did not complete the questionnaire.

### 3.1. Spirometric Survey

The spirometric test was performed on 200 subjects. Airflow obstruction (FEV_1_/FVC < 70%) was detected in 12 patients, who underwent a bronchodilator test as to the protocol; chronic airflow obstruction was diagnosed in 9 out of these 12 patients, according to the persistence of a FEV_1_/FVC < 70% after the test. In these 9 subjects, the severity of chronic airflow obstruction was rated moderate in 6 and mild in 3 subjects. Airflow obstruction was no longer detectable after the bronchodilator test in 3 of the 12 patients. In subjects without airflow obstruction, we observed a mean FEV_1_ of 105.4% pred ± 16.2, a mean FVC of 107.1% pred ± 14.5 and a mean FEV_1_/FVC of 81.9% pred ± 7.3 whereas in those with persistent airflow obstruction after bronchodilator test we observed a mean FVC, FEV_1_ and FEV_1_/FVC of 89.8% pred ± 13.6, 72.1% pred ± 12.4 and 63.5% pred ± 5.6, respectively.

There was a 4.5% prevalence of chronic airflow obstruction observed in the sample. Subjects with chronic airflow obstruction were homogeneously distributed between the two municipalities (Viggiano: *n* = 6; Grumento Nova: *n* = 3), considering the existing size ratio between the two populations (2:1). The group consisted of men only and was related to the different smoking habits observed in the sample between men and women (12.6 pack-years for men vs. 5.3 for women, *p* = 0.003), as smoking is an important risk factor in the development of respiratory diseases, and to the different occupational exposure to chemical and physical agents (54.8% for men vs. 23.0% for women, *p* < 0.001).

### 3.2. Descriptive Analysis of Respiratory Function and Symptomatology

The descriptive analysis was performed to assess the differences of risk factor distribution between the two areas (Table 1).

The sample mean age was 46.17 ± 15.08. The mean BMI of the sample was 28.23 ± 5.00, the prevalence of normal (or underweight) and obese subjects was heterogeneous in the two areas (*p* = 0.049). The PA showed a greater prevalence of subjects who reported being occupationally exposed to abundant amounts of chemical–physical agents (dust, fumes or gas) (48.35% vs. 33.00%, *p* = 0.039). Wood heating was common in homes of subjects residing in the PA (54.04% vs. 42.00%, *p* = 0.003). The PA showed a major prevalence of both subjects who worked in industries (26.37% vs. 10.00%, *p* = 0.004) and subjects residing near the main road that runs through the valley (36.26% vs. 4.00%, *p* < 0.0001).

A descriptive analysis was also carried out to assess the presence of significant associations between the examined risk factors and the selected outcomes. The results (Table S11 in Supplementary Material) highlighted that older subjects, subjects with higher BMI and a higher number of packets per year smoked and subjects with cardiovascular diseases showed a major susceptibility related to respiratory symptoms.

### 3.3. Multivariate Analysis of Respiratory Function and Respiratory Symptomatology

Concerning the distance-based models, multivariate analyses in general showed a decreasing prevalence (OR < 1) associated with an increasing distance from the centroid of the plant for all the outcomes investigated (Table 2).

A lower prevalence of high-grade dyspnoea (has to stop to take a breath at normal gait on the level) was significantly associated with an increased distance from the plant (ORa = 0.48; 95%CI: 0.25–0.92). Given the size of the study sample and the exploratory nature of this study, as the first to be carried out on a crude oil first treatment plant, some associations with *p* > 0.05 are worth noting, including “respiratory allergic symptoms" associated or not associated with eye symptoms (ORa = 0.76, 95% CI: 0.57–1.02 and ORa = 0.79, 95% CI: 0.57–1.08, respectively).

Differences in the occurrence of the investigated outcome between the group of residents in the PA and the group of residents in the RA were also assessed (area-based models). The results are substantially in agreement with those observed from the distance-based approach, showing a higher prevalence in the area proximal to the plant for all the investigated outcomes. There was a higher prevalence for “cough not due to a common cold for some periods of the year” and, as observed in the distance-based approach, for “respiratory allergic symptoms associated with eye symptoms” (ORa = 2.49, 95% CI:1.02–6.11; ORa = 2.53, 95% CI:1.29–4.94, respectively). Other excesses were found for “respiratory allergic symptoms” (ORa = 1.77, *p* = 0.109), “bronchial asthma or asthmatic bronchitis” (ORa = 2.36, *p* = 0.160), “cough and sputum not due to common cold for some period of the year” (ORa = 1.76, *p* = 0.1.72), and “chronic bronchitis” (ORa = 2.72, *p* = 0.187). A higher prevalence of high-grade dyspnea was observed in the proximal area (ORa = 1.63), but the statistical significance evidenced in the distance-based approach was not observed in the area-based approach.

## 4. Discussion

This study examined the respiratory health impacts on adults living near a first crude oil treatment plant in the Val d’Agri, Italy. After adjusting for selected confounding factors, the findings suggested a higher prevalence in subjects living closer to the plant for most of the outcomes investigated, in particular for high-grade dyspnoea and respiratory allergic symptoms associated with eye symptoms. 

The study area was also characterized according to proximity, orography and wind direction, in order to define a proxy of exposure for the population living around the plant. The proximity approach has been frequently used in many influential epidemiological and public health studies, particularly in the assessment of health effects around petrochemical industrial sites [24,25,26]. In addition, the approach has been used by governmental environmental protection bodies in their policies, such as the United States Environmental Protection Agency [27].

The subjects diagnosed in our study with chronic airflow obstruction were all men, which is probably related both to the different smoking habits and occupational exposure to chemical agents observed between men and women in the sample. The analysis results regarding respiratory function and symptoms are in agreement with several studies from the literature such as D’Amato et al. [28] and Baldacci et al. [29]. The 4.5% prevalence of COPD, a chronic inflammatory disease characterized by a limited respiratory airflow that cannot be completely reversed, is in agreement with national and international epidemiological estimates [9,30,31]. It is important to highlight that a COPD diagnosis requires a spirometry as this is an objective, reproducible and standardized measure of the airflow limitation. 

In agreement with our results, epidemiological studies have shown that living in the proximity of an industrial complex in general is associated with an increase in acute irritative symptoms of the respiratory tract and eye, and asthma [11,12], excess risk of respiratory diseases [13], acute respiratory problems [14], excess for hospitalization for asthma acute respiratory infection and COPD [15]. A study by Yang et al. was carried out in Taiwan to determine whether there was an excess of adverse respiratory and irritant health outcomes in populations living close to petrochemical manufacturing facilities [11]. Studies conducted among residents near the largest petrochemical industrial complex (60 petrochemical plants) in Thailand found an excess risk of respiratory diseases [13] and an association between acute respiratory problems and living closer (<5 km) to the complex [14]. A study conducted in the New York state examined whether living near a fuel-fired power plant increases the likelihood of hospitalization for respiratory diseases [15]. The authors observed significant increases in estimated rates of hospitalization for asthma, acute respiratory infection and COPD among individuals >10 years of age living in an area containing a fuel-fired power plant compared with an area with no power plant [15]. A study conducted in the Sohar Industrial Zone (Oman), containing a wide range of petrochemical industries and an iron smelter, found positive associations for all selected diseases (acute respiratory diseases, asthma, conjunctivitis and dermatitis) overall and stratified by gender and age groups [12] in the exposure zone, compared to the control zone.

In spite of the nature (crude oil first-treatment) and the size of the industrial site characterizing our study, our results were in line with the aforementioned studies reported in the scientific literature. In fact, from our survey, a significantly higher prevalence of allergic respiratory diseases (rhinitis and asthma) with an associated allergic ocular symptomatology was observed. We also believe that the statistically insignificant signals that were revealed by our study are worth noting, since the limited size of the study sample affects the statistical strength of the association. Several studies that have investigated the interaction between pollution and respiratory allergopathies suggest that damage to the respiratory mucosa and the defective mucociliary clearance induced by pollution can facilitate access to the immune system of the inhaled allergens [32]. This can increase the risk of atopic sensitization and the exacerbation of symptoms in sensitized subjects [33]. The current knowledge on allergy and asthma pathogenesis caused by the combined exposure to pollutants and allergens is principally based on in vitro or in vivo studies, because it is more difficult to study this interaction in the human population [34,35].

A few limitations of the present study need highlighting. This is a cross-sectional study where exposure and outcome are simultaneously assessed, so the evidence of a temporal relationship between them cannot be assessed and it is not possible to establish a true cause-and-effect relationship. In this study individual exposure was not assessed. Distance is a crude measure of exposure that can lead to some exposure misclassification. Moreover, the questionnaire included only questions about the occupational exposure in general, therefore no information was available on those 379 who work at the plant. There were also differences in confounders for subjects living in the proximal and in the reference area, so even adjusting for those confounders, the observed risk might be affected by residual confounding. The major drawback of our study is the reduced sample size, although some positive elements include the stratification of the sample, the high acceptance rate, and adjustments for several co-factors as described in the previous sections. The findings of this study might be affected by a multiple test problem even if a modest number of comparisons were performed.

Finally, the information regarding the respiratory symptoms was self-reported and subject to recall bias. However, some questions related to risk perception were included in the questionnaire and no differences in perception were detected between the residents of the two areas. In particular, no differences were observed between RA and PA, respectively, for the perception of personal exposure to air pollution (38 vs. 38.1%, *p* = 0.989), perception of a potentially health-threatening environment in the area of residence (65.7 vs. 67%, *p* = 0.841), willingness to move from the area of residence (54.0 vs. 48.4%, *p* = 0.435), perception of the risk of allergies in a polluted area (75.3 vs. 75%, *p* = 0.967), perception of the risk of acute respiratory diseases in a polluted area (84.1 vs. 77.6%, *p* = 0.292), and the perception of the risk of chronic respiratory diseases in a polluted area (79.8 vs. 75.0%, *p* = 0.464). These results strengthen our belief that the self-reported symptomatology was not substantially influenced by recall bias.

## 5. Conclusions

This study shows a higher prevalence for subjects living closer to the largest crude oil first treatment plant in Italy for most of the investigated outcomes and particularly for high-grade dyspnoea and respiratory allergic symptoms associated with eye symptoms.

Despite the limitations previously described, the merits of this study are (i) the uniqueness of the investigation on respiratory symptoms in an area characterized by a crude oil first treatment plant; (ii) the overlapping of the results for the distance and the area-based analysis; and (iii) that the evidence was obtained after adjustment for several risk factors. 

As this is the first environmental epidemiologic study carried out regarding a crude oil first treatment plant, these findings contribute to increasing awareness and further research on environmental health issues. These findings will hopefully encourage the initiation of preventive actions and public health intervention programs in Italy. In addition, our findings highlight the need for a space–time surveillance system in the entire area of the two municipalities. Before the definition and calibration of this surveillance system, the study should be repeated with a larger sample, including the subjects already studied.

## Figures and Tables

**Table 1:** Descriptive analysis of risk factors according to the residence area (proximal versus reference).

Variable	Reference Area		Proximal Area		Total		*p*
	*n*	% (or mean ± SD)	*n*	% (or mean ± SD)	*n*	% (or mean ± SD)	
Gender							1.000
Men	54	54.00	50	54.95	104	54.45	
Women	46	46.00	41	45.05	87	45.55	
Total	100	100.00	91	100.00	191	100.00	
Age	100	45.13 ± 15.03	91	47.31 ± 15.13	191	46.17 ± 15.08	0.320
BMI (categorical)							0.049
Normal	34	34.00	19	20.88	53	27.75	
Overweight	42	42.00	37	40.66	79	41.36	
Obese	24	24.00	35	38.46	59	30.89	
Total	100	100.00	91	100.00	191	100.00	
BMI (continuous)	100	27.39 ± 4.60	91	29.16 ± 5.27	191	28.23 ± 5.00	0.014
Pack-years	98	9.43 ± 16.44	89	8.98 ± 17.84	187	9.22 ± 17.07	0.858
Occupational Exposure							0.039
NO	67	67.00	47	51.65	114	59.69	
YES	33	33.00	44	48.35	77	40.31	
Total	100	100.00	91	100.00	191	100.00	
Cardiovascular comorbidity							0.762
NO	67	67.00	59	64.84	126	65.97	
YES	33	33.00	3291	35.16	65	34.03	
Total	100	100.00		100.00	191	100.00	
Respiratory symptoms in childhood							0.221
NO	82	82.00	67	73.63	149	78.01	
YES	18	18.00	2491	26.37	42	21.99	
Total	100	100.00		100.00	191	100.00	
Family history of respiratory diseases							0.008
NO	72	72.00	70	76.92	142	74.35	
YES	28	28.00	21	23.08	49	25.65	
Total	100	100.00	91	100.00	191	100.00	
Wood heating							0.008
NO	58	58.00	32	35.96	90	47.62	
YES	42	42.00	57	64.04	99	52.38	
Total	100	100.00	89	100.00	189	100.00	
Employed							0.083
NO	50	50.00	34	37.36	84	43.99	
YES	50	50.00	57	62.64	107	56.02	
Total	100	100.00	91	100.00	191	100.00	
Metabolic comorbidity							1.000
NO	72	72.00	66	72.53	138	43.98	
YES	28	28.00	25	27.47	53	56.02	
Total	100	100.00	91	100.00	191	100.00	
Distance from the main road running through the valley (buffer 500 m)							<0.0001
Near	4	4.00	33	36.26	37	19.37	
Far	96	96.00	58	63.74	154	80.63	
Total	100	100.00	91	100.00	191	100.00	
Employed in Industry							0.004
NO	90	90.00	67	73.63	157	82.20	
YES	10	10.00	24	26.37	34	17.80	
Total	100	100.00	91	100.00	191	100.00	

Notes—N: number; SD: standard deviation; BMI: body mass index. Acute myocardial infarction or ischemic heart disease, stroke or transient ischemic attack, obliterative arterial disease of the lower limbs, deep vein thrombosis, and embolism were considered as cardiovascular comorbidity. Jaundice, liver disorders or colic, liver failure, hepatitis, high cholesterol and/or triglycerides in the blood, diabetes were considered as metabolic comorbidities.

**Table 2:** Multivariate analysis with logistic regression, related to the distance-based approach. Odds ratios are expressed per 1 km distance of place of residence from the plant.

Outcomes	OR	*p*	95% CI	ORa	*p*	95% CI
Cough (not due to a common cold) for some periods of the year	0.74	0.070	0.53–1.02	0.79	0.215	0.54–1.14
Cough ( not due to a common cold) for some periods of the year and for at least 2 years	0.76	0.172	0.51–1.13	0.81	0.331	0.53–1.24
Cough and sputum (not due to a common cold) for some periods of the year	0.83	0.201	0.62–1.11	0.90	0.528	0.65–1.24
Cough and sputum (not due to a common cold) for some periods of the year and for at least 2 years	0.82	0.264	0.59–1.16	0.87	0.465	0.61–1.26
High–grade dyspnoea (has to stop to take a breath at normal gait on the level)	0.51	0.015	0.30–0.88	0.48	0.027	0.25–0.92
Chronic bronchitis	0.97	0.897	0.59–1.58	0.82	0.442	0.49–1.37
Bronchial asthma or asthmatic bronchitis	0.83	0.386	0.55–1.26	0.79	0.337	0.48–1.28
Respiratory allergic symptoms associated with eye symptoms	0.76	0.035	0.60–0.98	0.76	0.067	0.57–1.02
Respiratory allergic symptoms	0.81	0.126	0.63–1.06	0.79	0.143	0.57–1.08

Notes—OR: odds ratio; ORa: adjusted odds ratio; *p*: *p*-value; 95% CI: 95% confidence interval. “Chronic bronchitis”: when a participant stated that he/she had been diagnosed with chronic bronchitis. “Respiratory allergic symptoms” are a combination of (i) hay fever or some other condition that causes rhinorrhea or stuffy nose outside of common colds, (ii) attacks of breathing difficulty with wheezing (not due to a common cold), (iii) allergic reactions to powder, synthetic fibers, feathers and hairs. “Eye symptoms”: pruritus, redness and burning. Adjustments were performed for gender, age, BMI, smoking, cardiovascular comorbidity, occupational exposure, exposure to chemical–physical agents, and distance from the main road (buffer of 500 m). In the case of bronchial asthma and allergic symptoms, adjustment was performed for gender, age, BMI (for bronchial asthma only), smoking, occupational exposure, exposure to chemical-physical agents, and distance from the main road (buffer of 500 m).

## Supplementary Materials

The following are available online at http://www.mdpi.com/1660-4601/15/12/2636/s1, Figure S1: Distribution of residents in the municipalities of Viggiano and Grumento Nova between 17–73 years. In light blue, subjects residing in the PA. In dark blue, subjects residing in the RA. Figure S2: Distribution of the sample of the respiratory function study extracted from residents in the municipalities of Viggiano and Grumento Nova aged between 17–73, identifying subjects in the PA (yellow) and subjects in the RA (light green). Table S1: Subjects aged 17–73 residing in Viggiano and Grumento Nova municipalities. Table S2: Gender of subjects aged 17–73 residing in Viggiano and Grumento Nova municipalities. Table S3: Age classes of subjects aged 17–73 residing in Viggiano and Grumento Nova municipalities. Table S4: Subjects aged 17–73 residing in Viggiano and Grumento Nova by municipality. gender and age classes. Table S5: Distribution by municipality, gender and tertile of age of residents, aged 17–73, in the municipalities of Viggiano and Grumento Nova, classified as resident in the PA. Table S6: Distribution by municipality, gender and tertile of age of residents, aged 17–73 and classified as resident in the PA, of the sample for the study on respiratory function. Table S7: Distribution by municipality, gender and tertile of age of residents, aged 17–73, in the municipalities of Viggiano and Grumento Nova, classified as resident in the RA. Table S8: Distribution by municipality, gender and tertile of age of residents, aged 17–73 and classified as resident in the RA, of the sample for the study on respiratory function. Table S9: Distribution by municipality, gender and residence area of the 191 subjects of the sample used for the descriptive and multivariate analyses. Table S10: Results of the spirometry test. Table S11: Results of the descriptive analysis risk factors versus outcome.
