# Supplementary material for: Respiratory Symptoms in Relation to Living near a Crude Oil First Treatment Plant in Italy: A Cross-Sectional Study
Source: Int J Environ Res Public Health. 2018 Nov 25;15(12):2636. doi: 10.3390/ijerph15122636 (PMC6313728; doi:10.3390/ijerph15122636)
Supplement: Supplementary file 1 [file ijerph-15-02636-s001.pdf]

Supplementary Material

Respiratory Symptoms in Relation to Living Near a Crude Oil First Treatment Plant in Italy: A Cross-Sectional Study

Elisa Bustaffa [1,§], Alessio Coi [1,§], Fabrizio Minichilli [1], Michele Santoro [1], Renato Prediletto [2], Simonetta Monti [2], Ivana Pavlickova [2] and Fabrizio Bianchi [1,*]

Method for the sample extraction

From the municipal registries of the two municipalities, 3,641 residents were selected (at 31th December 2014) aged 17-73, divided by gender, age class and residential municipality (Tables S1-S4).

Table S1. Subjects aged 17-73 residing in Viggiano and Grumento Nova municipalities.

Municipality	Number	%
**Viggiano**	2393	65.7
**Grumento Nova**	1248	34.3
TOTAL	3641	100

Table S2. Gender of subjects aged 17-73 residing in Viggiano and Grumento Nova municipalities.

Gender	Number	%
**Men**	1833	50.3
**Women**	1808	49.7
TOTAL	3641	100

Table S3. Age classes of subjects aged 17-73 residing in Viggiano and Grumento Nova municipalities.

Age class	Number	%
**17-26**	563	15.5
**27-36**	735	20.2
**37-46**	675	18.5
**47-56**	776	21.3
**57-66**	653	17.9
**67-73**	239	6.6
TOTAL	3641	100.00

Table S4. Subjects aged 17-73 residing in Viggiano and Grumento Nova municipalities by municipality. gender and age classes.

	Men						Women					
**Age class**	**17-26**	**27-36**	**37-46**	**47-56**	**57-66**	**67-73**	**17-26**	**27-36**	**37-46**	**47-56**	**57-66**	**67-73**
**Viggiano**	192	254	251	259	193	64	194	247	205	266	192	76
**Grumento Nova**	87	130	102	123	124	54	90	104	117	128	144	45
TOTAL	279	384	353	382	317	118	284	351	322	394	336	121

The sampling was performed several months before the spirometry, in order to avoid to do the spirometric test in those seasons in which the incidence of influenzae and of allergic episodes is higher. Sampling selection referred to an age range 17-73 ensuring the recruitment of a final sample to be submitted to spirometric tests in the age range 18-74.

Subjects residing in the proximal area (PA) were 529 and those residing in the reference area (RA) were 3,112 (Figure S1). The PA and RA boundaries do not match with the municipal ones. As reported in the manuscript PA and RA were defined by an ellipse of major and minor axis of 12 and 4 km, respectively, centered on the plant.

Figure S1. Distribution of residents in the municipalities of Viggiano and Grumento Nova in the range of 17-73 years. In light blue subjects residing in the PA. In dark blue subjects residing in the RA.

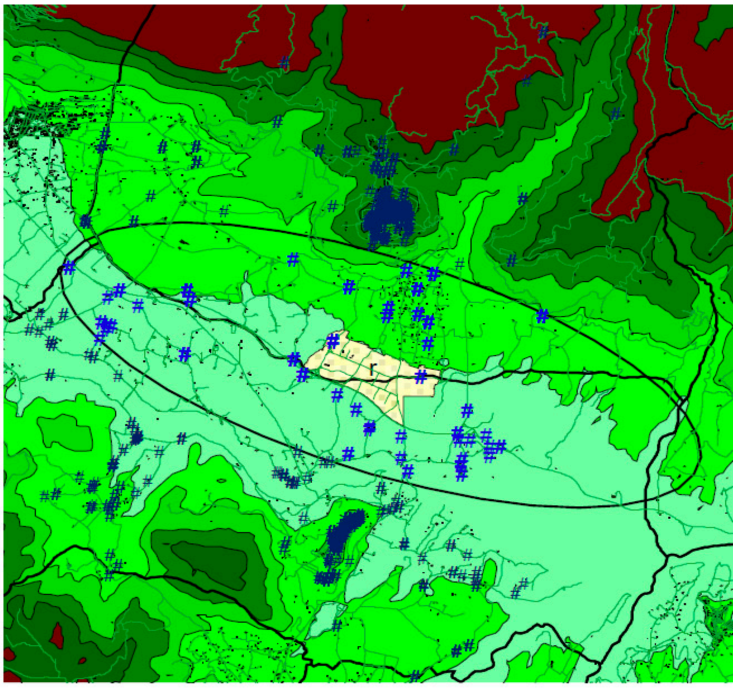

Subjects residing in the PA were divided in age classes, defined considering the tertiles of age, gender and municipality (Table S5).

Table S5. Distribution by municipality, gender and tertile of age of residents, aged 17-73, in the municipalities of Viggiano and Grumento Nova, classified as resident in the PA.

	Men				Women			
**Age class**	**17-35**	**36-53**	**54-73**	*Total*	**17-35**	**36-53**	**54-73**	*Total*
**Viggiano**	63(11.9)	63 (11.9)	56 (10.6)	182	58 (10.9)	49 (9.3)	44 (8.3)	151
**Grumento Nova**	30 (5.7)	33 (6.2)	39(7.4)	102	26 (4.9)	31 (5.9)	37 (7.0)	94
**TOTAL**	93	96	95	284	84	80	81	245

Note - In brackets is shown the percentage with respect to the 529 subject residing in the PA.

Therefore, 120 subjects residing in the PA (22.7% on 529) were randomly extracted on the basis of the distribution for municipality-gender-age class. The entity of the sample in each cell was re-calculated distributing the 120 subjects to be extracted on the basis of percentages reported in Table 6.

Table S6. Distribution by municipality, gender and tertile of age of residents, aged 17-73 and classified as resident in the PA, of the sample for the study on respiratory function.

	Men				Women			
**Age class**	**17-35**	**36-53**	**54-73**	*Total*	**17-35**	**36-53**	**54-73**	*Total*
**Viggiano**	14	14	13	41	13	11	10	34
**Grumento Nova**	7	8	9	24	6	7	8	21
**TOTAL**	21	22	22	65	19	18	18	55

Subjects residing in the RA were divided by age classes, defined considering the tertiles of age, gender and municipality (Table S7).

Table S7. Distribution by municipality, gender and tertile of age of residents, aged 17-73, in the municipalities of Viggiano and Grumento Nova, classified as resident in the RA.

	Men				Women			
**Age class**	**17-35**	**36-53**	**54-73**	*Total*	**17-35**	**36-53**	**54-73**	*Total*
**Viggiano**	355(11.4)	365 (11.7)	311 (10.0)	1031	354 (11.4)	352 (11.3)	323 (10.4)	1029
**Grumento Nova**	171 (5.5)	150 (4.8)	197(6.3)	518	158 (5.1)	170 (5.9)	206 (7.0)	534
**TOTAL**	526	515	508	1549	512	522	529	1563

Note - In brackets is shown the percentage with respect to the 3112 subject residing in the RA.

Distribution by age, gender and municipality of subject residing in the RA is similar to that of the residents in the PA. This allowed to extract a sample that reflects the age-gender-municipality distribution of the residents of both the areas (frequency matching).

The numerosity of the extracted sample of subjects residing in in theR is the same of the that of those residing in the PA (120 subjects represent the 3.6% of the total of the subjects residing outside the PA). The sample was randomly extracted for each municipality-gender-age class cell. The numerosity of the sample of each cell was calculated re-distributing the 120 subjects to be extracted on the basis of percentages reported in the previous table. The extracted sample is reported in Table S8.

Table S8. Distribution by municipality, gender and tertile of age of residents, aged 17-73 and classified as resident in the RA, of the sample for the study on respiratory function.

	Men				Women			
**Age class**	**17-35**	**36-53**	**54-73**	***Total***	**17-35**	**36-53**	**54-73**	***Total***
**Viggiano**	14	14	12	40	13	13	12	38
**Grumento Nova**	7	6	8	21	6	7	8	21
**TOTAL**	21	20	20	61	19	20	20	59

Table S9 shows distribution by municipality, gender and residence area of the analyzed sample.

Table S9. Distribution by municipality, gender and residence area of the 191 subjects of the sample used for the descriptive and multivariate analysis.

Municipality		Men		Women		Men+Women		Men+Women
		PA	RA	PA	RA	PA	REA	Total
**Viggiano**	**Selected**	41	40	34	38	75	78	**153**
	**Study sample**	34	35	23	32	57	67	**124**
**Grumento Nova**	**Selected**	24	21	21	21	45	42	**87**
	**Study sample**	16	19	18	14	34	33	**67**
**Total**	**Selected**	65	61	55	59	120	120	**240**
	**Study sample**	50	54	41	46	91	100	**191**

Notes: PA: Proximal Area; RA: Reference Area.

Figure S2 shows all the residents. Samples extracted are representative of the spatial distribution within the two sampling areas, adopting a different fraction of sampling aimed at ensuring a greater number of subjects in the PA.

Figure S2. Distribution of the sample of the respiratory function study extracted from residents in the municipalities of Viggiano and Grumento Nova ages 17-73, identifying subjects in the PA (yellow) and subjects in the RA (light green).

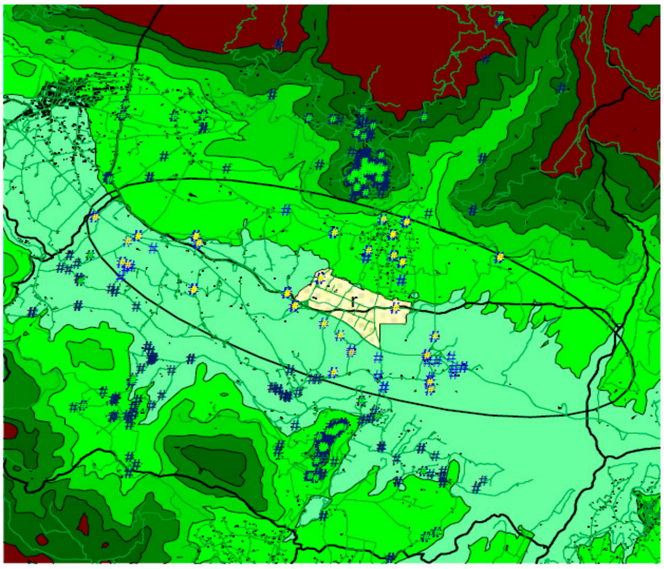

Spirometry results

Table S10. Results of the spirometry test.

PERSONAL DATA							*SPIROMETRY*							
IND_COD	*DATE*	*AGE (years)*	*HEIGHT (cm)*	*WEIGHT (kg)*	*SEX*	*BMI (kg/m^2)*	*FVC (L)*	*FEV_1 (L)*	*FEV_1/FVC %*	*FEV25-75% (L/sec)*	*PEF (L/sec)*	*FEF25% (L/sec)*	*FEF50% (L/sec)*	*FEF75% (L/sec)*
VF133	02/12/16	47	159	73	F	28.88	111	102	79	73	90	100	122	50
VF124	02/12/16	22	158	87	F	34.85	116	99	74	59	94		67	
GM55	02/12/16	74	160	90	M	35.16	81	72	67	40	60	40	38	56
GF23	02/12/16	69	146	65	F	30.49	143	153	87	96	125	125	97	111
GF5	02/12/16	42	160	63	F	24.61	128	127	85	115	122	132	146	99
VM188	02/12/16	47	160	55	M	21.48	95	95	83	73	94	108	94	61
GF27	02/12/16	25	158	60	F	24.03	110	112	89	94	84	88	99	99
VM108	02/12/16	62	168	107	M	37.91	88	105	93	140	104	108	154	153
GF29	02/12/16	55	162	55	F	20.96	108	103	81	81	89	89	80	90
VM125	02/12/16	61	163	91	M	34.25	76	59	62	24	79	69	24	23
GM36	03/12/16	61	162	108	M	41.15	111	119	85	127	99	100	141	144
GM21	03/12/16	55	171	69	M	23.60	122	123	81	121	99	91	144	97
VF6	03/12/16	30	151	55	F	24.12	102	102	86	91	105	92	107	80
GM71	03/12/16	60	168	99	M	35.08	93	93	79	79	66	63	83	86
VF83	03/12/16	51	145	65	F	30.92	105	111	88	106	104	108	116	106
VF102	03/12/16	38	156	55	F	22.60	129	126	84	111	122	135	132	88
VM28	03/12/16	22	168	67	M	23.74	132	139	90	115	80	93	114	157
VF72	03/12/16	57	159	75	F	29.67	129	133	87	128	116	130	158	123
GM89	03/12/16	55	177	81	M	25.85	114	117	82	117	122	139	130	86
VM149	04/12/16	35	174	119	M	39.31	105	106	84	103	141	164	129	80
VM131	04/12/16	57	162	65	M	24.77	141	145	82	136	141	157	179	114
VF98	04/12/16	49	160	53	F	20.70	119	118	85	103	114	128	105	110
VM89	04/12/16	32	173	75	M	25.06	107	98	76	70	93	94	76	66

VM85	04/12/16	23	174	85	M	28.08	115	119	88	127	105	123	141	130
VF65	04/12/16	61	163	73	F	27.48	111	101	76	68	94	105	85	51
GF28	04/12/16	41	156	64	F	26.30	123	120	84	97	91	92	113	90
GM38	04/12/16	59	168	73	M	25.86	116	122	84	134	139	153	180	101
GF43	04/12/16	35	151	122	F	53.51	100	98	85	80	112	117	103	61
GM8	04/12/16	37	172	85	M	28.73	127	125	82	116	126	145	141	98
VM117	05/12/16	48	177	94	M	30.00	119	117	80	96	142	161	108	78
VM71	05/12/16	32	163	49	M	18.44	91	102	96	95	59	68	93	169
VF146	05/12/16	62	158	72	F	28.84	122	121	83	106	122	133	119	82
GF77	05/12/16	39	150	59	F	26.22	109	109	85	87	89	96	102	83
VF49	05/12/16	41	165	83	F	30.49	99	88	77	62	76	85	67	53
VF11	07/12/16	25	155	52	F	21.64	104	100	84	76	63	63	76	109
VF80	07/12/16	57	171	85	F	29.07	118	108	77	84	102	110	106	59
GF40	07/12/16	46	157	78	F	31.64	101	109	92	93	104	110	91	118
VM96	07/12/16	28	171	74	M	25.31	103	102	84	93	121	136	103	82
GM20	07/12/16	28	178	76	M	23.99	104	110	89	128	100	118	153	133
VF99	07/12/16	35	165	76	F	27.92	109	109	87	112	115	121	116	110
GF42	07/12/16	31	176	75	F	24.21	121	102	74	74	82	82	86	62
GM47	07/12/46	74	162	70	M	26.67	95	101	80	92	75	79	110	92
GF37	09/12/16	19	160	67	F	26.17	119	117	86	104	99	111	107	95
VF26	09/12/16	35	162	73	F	27.82	127	122	83	116	122	139	155	86
GM50	09/12/16	74	162	78	M	29.72	86	93	83	90	81	88	108	84
GM64	09/12/16	27	181	72	M	21.98	111	119	90	127	101	118	128	134
GF75	09/12/16	72	145	65	F	30.92	141	154	87	146	98	91	138	188
GF87	09/12/16	32	165	63	F	23.14	113	110	84	97	113	122	108	86
GF57	09/12/16	62	155	59	F	24.56	116	112	81	84	115	127	118	68
VM126	09/12/16	55	171	77	M	26.33	107	102	76	75	100	114	96	52
GM51	09/12/16	46	179	95	M	29.65	113	100	72	62	114	130	74	43
VM136	09/12/16	66	172	95	M	32.11	93	76	63	41	83	48	40	40
GM65	09/12/16	37	177	97	M	30.96	105	120	94	164	121	140	167	211
GM32	10/12/16	61	163	73	M	27.48	103	99	77	69	100	96	78	71
GF60	10/12/16	58	161	77	F	29.71	114	103	77	60	85	76	57	65
GF80	10/12/16	30	153	53	F	22.64	105	101	84	81	94	102	94	69
GM69	10/12/16	69	162	76	M	28.96	105	109	80	95	131	146	117	76
GF82	10/12/16	59	151	75	F	32.89	127	134	88	135	93	96	133	166
GM31	10/12/16	24	166	74	M	26.85	104	97	80	69	111	114	76	73
GF93	10/12/16	58	148	54	F	24.65	103	120	97	113	103	101	116	143
GM06	12/12/16	28	175	74	M	24.16	88	86	82	79	59	64	93	75
VF129	12/12/16	61	150	74	F	32.89	115	110	79	69	115	109	90	47
VF95	12/12/16	43	157	62	F	25.15	150	138	79	98	108	111	112	88
GM85	12/12/16	38	165	65	M	23.88	113	111	83	95	98	112	103	96
GF56	12/12/16	68	151	89	F	39.03	104	101	79	54	83	82	66	42
VF48	12/12/16	23	167	71	F	25.46	99	93	82	71	105	114	74	65
VM106	14/12/16	31	174	126	M	41.62	97	98	85	106	130	149	155	75
VF121	14/12/16	28	175	67	F	21.88	103	100	84	97	99	113	121	84
GF61	14/12/16	34	161	66	F	25.46	115	117	88	141	102	102	145	143
GM76	14/12/16	57	176	103	M	33.25	104	100	77	77	110	119	75	75
VM33	14/12/16	42	160	74	M	28.91	119	133	93	154	122	138	194	178
GF90	15/12/16	48	160	98	F	38.28	94	92	84	94	114	105	149	60
GM19	15/12/16	66	176	78	M	25.18	112	104	72	65	96	107	87	40
VF119	15/12/16	37	156	69	F	28.35	105	94	77	61	77	76	76	50
VF90	15/12/16	25	159	55	F	21.76	104	108	90	109	86	97	113	115
GM84	16/12/16	27	169	77	M	26.96	110	117	90	140	92	106	151	177
VM59	16/12/16	46	174	107	M	35.34	118	100	69	61	77	81	66	46
VF42	16/12/16	51	164	74	F	27.51	137	138	86	138	123	134	158	130
VM19	16/12/16	28	180	75	M	23.15	84	82	81	70	100	111	84	56
GM49	19/12/16	38	181	104	M	31.75	101	98	80	73	68	65	68	86
GM81	19/12/16	46	170	88	M	30.45	96	87	75	52	104	115	59	44
GF79	19/12/16	38	155	73	F	30.39	114	109	82	85	105	109	106	71
GM73	19/12/16	50	181	122	M	37.24	110	113	82	112	102	117	110	96
GF4	21/12/16	52	154	67	F	28.25	114	113	84	95	79	67	92	122
GM35	21/12/16	29	174	78	M	25.76	115	118	86	115	108	120	134	114
VF152	21/12/16	28	152	51	F	22.07	101	106	91	94	87	91	103	106
VM93	21/12/16	63	167	79	M	28.33	108	104	76	76	100	85	82	70
GM83	21/12/16	50	171	96	M	32.83	91	73	65	40	89		40	
GF70	22/12/16	54	153	63	F	26.91	66	72	92	85	89	78	141	59
VM105	22/12/16	48	180	100	M	30.86	99	94	76	72	120	125	72	59
VM104	22/12/16	59	167	84	M	30.12	83	70	67	36	97	89	41	30
GM92	22/12/16	30	174	78	M	25.76	105	94	75	61	129	91	76	50
GF91	22/12/16	25	158	61	F	24.44	127	122	84	104	102	114	123	91
VM1	22/12/16	40	172	76	M	25.69	90	98	90	117	89	65	124	168
VM17	22/12/16	26	180	69	M	21.30	100	101	85	98	105	123	115	94
GM59	22/12/16	29	171	83	M	28.38	109	104	81	88	82	96	109	81
VM151	22/12/16	49	171	107	M	36.59	99	88	72	56	90	86	69	52
GM63	22/12/16	20	166	88	M	31.93	96	85	76	59	68	71	69	60

VF140	23/12/16	27	157	63	F	25.56	116	98	74	55	104	104	62	48
GF13	23/12/16	62	149	53	F	23.87	110	99	74	52	66	46	66	43
VM45	30/12/16	46	175	80	M	26.12	98	107	89	149	100	113	142	181
GM48	30/12/16	53	160	80	M	31.25	127	135	87	122	69	78	114	219
VM15	18/10/16	18	166	75	M	27.22	135	113	72	72	93		79	
VF7	18/10/16	44	158	53	F	21.23	117	118	86	119	118		140	
VM20	18/10/16	39	177	85	M	27.13	107	104	80	85	124		99	
VM4	18/10/16	60	160	70	M	27.34	113	113	80	90	151		122	
GF25	18/10/16	40	151	88	F	38.59	95	96	87	83	85		114	
VM12	15/11/16	65	172	98	M	33.13	97	99	80	88	128	144	95	72
VF8	15/11/16	55	165	89	F	32.69	101	91	77	68	113	119	107	42
VM21	15/11/16	70	171	78	M	26.67	105	108	79	105	102	107	135	72
VM23	15/11/16	72	165	81	M	29.75	84	69	63	33	68	58	29	33
VF24	15/11/16	65	155	61	F	25.39	108	117	90	152	124	119	189	155
VM30	15/11/16	65	175	93	M	30.37	91	101	86	136	107	118	145	124
VF35	15/11/16	60	160	82	F	32.03	91	90	83	78	88	95	85	66
VF36	15/11/16	41	155	95	F	39.54	98	95	83	74	108	120	85	64
VM34	15/11/16	64	168	90	M	31.89	111	116	82	108	90	100	126	108
VF145	16/11/16	28	153	55	F	23.50	98	100	89	97	88	86	108	98
VM5	16/11/16	42	181	87	M	26.56	112	113	82	109	108	115	132	91
VF143	16/11/16	45	156	51	F	20.96	119	123	88	118	118	110	137	121
VF137	16/11/16	68	158	74	F	29.64	104	108	87	92	108	117	92	118
VM142	16/11/16	69	165	80	M	29.38	80	78	75	53	89	99	58	46
VM39	16/11/16	39	172	96	M	32.45	102	106	86	102	128	143	107	108
VF147	16/11/16	38	156	76	F	31.23	104	107	89	123	108	120	151	112
VF44	16/11/16	64	158	79	F	31.65	126	117	77	77	118	92	93	67
VF141	16/11/16	60	150	64	F	28.44	124	123	83	90	107	107	121	78
VM120	17/11/16	59	171	84	M	28.73	136	127	74	86	147	156	104	63
VM134	17/11/16	43	170	74	M	25.61	118	123	85	140	128	130	181	114
VM144	17/11/16	70	167	70	M	25.10	88	96	84	116	93	73	126	109
VM118	17/11/16	55	162	93	M	35.44	99	95	78	76	76	67	107	54
VF92	17/11/16	50	163	94	F	35.38	120	111	79	81	131	143	95	69
GM46	17/11/16	53	174	80	M	26.42	109	89	65	50	86	68	52	43
VF135	17/11/16	34	160	86	F	33.59	102	105	88	129	141	153	166	110
GF53	17/11/16	57	156	70	F	28.76	128	127	83	105	102	103	135	88
VF115	17/11/16	24	156	63	F	25.89	115	110	83	93	109	121	131	71
GF2	27/11/16	64	158	98	F	39.26	82	77	78	52	98		78	
VM56	27/11/16	29	167	79	M	28.33	119	124	88	124	86	85	128	175
GM54	27/11/16	71	175	83	M	27.10	96	100	80	92	101	106	98	77
VM53	27/11/16	60	172	64	M	21.63	102	104	73	69	98	109	89	48
VF43	27/11/16	20	165	68	F	24.98	88	93	91	94	74	87	110	119
VF29	27/11/16	34	150	54	F	24.00	116	113	84	88	106	115	120	74
VM69	27/11/16	28	185	85	M	24.84	110	111	84	109	117	131	116	95
VF47	27/11/16	56	155	66	F	27.47	105	105	84	85	92	96	108	69
VF58	27/11/16	51	159	92	F	36.39	113	102	76	65	92	95	84	43
GM12	27/11/16	42	181	73	M	22.28	112	100	73	73	74	84	78	55
VF75	29/11/16	66	157	70	F	28.40	98	94	80	62	94	104	73	43
VM66	29/11/16	69	153	57	M	24.35	140	129	72	66	127	141	96	56
VM79	29/11/16	62	171	104	M	35.57	95	98	81	86	113	123	80	90
VF78	29/11/16	43	163	92	F	34.63	101	92	79	64	108	107	76	56
VM64	29/11/16	73	175	88	M	28.73	91	59	49	26	48	30	22	42
GF88	29/11/16	56	155	92	F	38.29	112	104	78	67	106	106	81	54
VF74	29/11/16	47	160	59	F	23.05	122	118	83	93	136	150	107	81
VM62	29/11/16	46	188	101	M	28.58	95	95	80	95	85	96	119	67
GF11	29/11/16	27	160	63	F	24.61	104	106	89	113	98	98	121	109
VM67	29/11/16	35	176	90	M	29.05	101	102	83	92	108	122	97	93
GF16	30/11/16	73	154	86	F	36.26	85	91	88	78	82	73	80	86
VM76	30/11/16	19	180	79	M	24.38	107	117	91	156	107	113	172	166
VM77	30/11/16	51	173	112	M	37.42	108	104	78	85	99	108	103	63
GM17	30/11/16	51	177	98	M	31.28	98	104	85	151	130	140	198	109
VF38	30/11/16	45	160	54	F	21.09	99	95	82	79	72	78	89	77
VM55	30/11/16	52	175	84	M	27.43	107	101	76	74	120	130	90	66
GM18	30/11/16	58	174	88	M	29.07	119	124	83	132	112	106	138	120
VF84	30/11/16	57	156	58	F	23.83	124	121	82	95	110	116	121	81
VF73	30/11/16	26	166	103	F	37.38	93	91	85	90	97	99	104	75
VM81	30/11/16	50	171	84	M	28.73	91	95	84	98	110	126	121	79
VM100	01/12/16	45	180	85	M	26.23	118	104	71	73	78		79	
VM60	01/12/16	51	170	106	M	36.68	106	103	78	81	94	101	109	68
GM22	01/12/16	45	179	63	M	19.66	106	103	79	90	77	89	85	92
VM86	01/12/16	38	171	82	M	28.04	105	109	86	103	141	158	63	125
VF87	01/12/16	69	171	68	F	23.26	94	90	80	80	63	66	90	79
VF10	18/11/16	55	167	74	F	26.53	97	76	67	38	82	66	42	31
VF82	18/11/16	60	147	79	F	36.56	122	127	87	107	99	105	119	118
VM109	18/11/16	37	187	138	M	39.46	144	130	79	113	136	163	115	90
VM132	19/11/16	36	168	71	M	25.16	83	75	75	48	85		56	

VM107	19/11/16	50	162	88	M	33.53	89	91	84	82	84	91	90	90
VF2	19/11/16	66	148	60	F	27.39	117	132	93	132	83	78	133	190
VM139	20/11/16	23	174	63	M	20.81	81	89	93	99	96	110	103	124
VM148	20/11/16	46	164	61	M	22.68	104	100	80	83	109	126	100	61
VM113	20/11/16	34	175	82	M	26.78	119	108	76	79	122	116	87	63
VM94	20/11/16	37	174	86	M	28.41	107	117	90	132	97	112	139	157
VF70	20/11/16	52	144	48	F	23.15	137	130	79	76	96	101	102	62
VM138	20/11/16	28	170	63	M	21.80	106	108	86	100	118	128	98	118
VM114	20/11/16	23	183	70	M	20.90	101	99	82	87	105	102	91	84
VF128	21/11/16	57	159	99	F	39.16	89	103	98	118	65	46	102	237
GF1	21/11/16	38	157	65	F	26.37	125	121	83	99	113	111	120	86
GM66	21/11/16	31	171	76	M	25.99	115	114	83	118	98	113	156	90
GF33	21/11/16	64	157	78	F	31.64	89	92	87	77	70	73	77	89
VM18	22/11/16	37	168	98	M	34.72	105	110	88	106	93	84	107	129
VM112	22/11/16	31	188	85	M	24.05	118	114	97	99	105	114	103	87
GM30	22/11/16	72	170	82	M	28.37	69	62	68	35	63	52	36	46
GM3	22/11/16	62	169	60	M	21.01	105	106	79	96	77	86	123	67
GF14	22/11/16	15	169	68	F	23.81	112	107	83	95	105	119	103	88
VM26	22/11/16	51	161	86	M	33.18	86	60	57	22	87	45	22	25
VF32	22/11/16	48	156	88	F	36.16	96	94	84	76	112	110	108	63
VF52	22/11/16	28	155	45	F	18.73	113	113	87	105	102	111	131	96
VF51	22/11/16	24	157	55	F	22.31	117	113	85	97	84	83	108	93
VM68	22/11/16	22	169	73	M	25.56	94	104	94	117	90	106	126	148
VF150	23/11/16	35	156	64	F	26.30	111	117	90	138	102	114	141	177
VM37	23/11/16	41	182	96	M	28.98	83	89	88	136	121	132	171	109
VM130	23/11/16	32	170	92	M	31.83	85	92	91	132	89	103	147	147
VM31	23/11/16	38	178	81	M	25.56	106	112	88	133	100	108	154	129

Legend – IND_COD: individual code; BMI: Body Mass Index; FVC: Forced Vital Capacity; FEV_1: Forced Expiratory Volume in one second; FEV25-75%: Forced Expiratory Volume from 25% to 75% of vital capacity; PEF: Peak Expiratory Flow; FEF25%: Forced Expiratory Flow at 25% of vital capacity; FEF50%: Forced Expiratory Flow at 50% of vital capacity; FEF75%: Forced Expiratory Flow at 75% of vital capacity.

Descriptive analysis Vs outcome

Table S11: Results of the descriptive analysis risk factors versus outcome.

		Cough (not due to a common cold) for some periods of the year							Cough (not due to a common cold) for some periods of the year and for at least 2 years							Cough and sputum (not due to a common cold) for some period of the year and for at least 2 years						
		NO		YES		TOTAL			NO		YES		TOTAL			NO		YES		TOTAL		
		N	% or mean	N	% or mean	N	% or mean	p	N	% or mean	N	% or mean	N	% or mean	p	N	% or mean	N	% or mean	N	% or mean	p
	**M=0**	90	57.69	14	40.00	104	54.45		94	55.62	10	45.45	104	54.45		102	55.74	2	28.57	104	54.74	
**Sex**	**F=1**	66	42.31	21	60.00	87	45.55		75	45.45	12	54.55	87	45.55		81	44.26	5	71.43	86	45.26	
	**Total**	156	100.00	35	100.00	191	100	0.063	169	101.07	22	100.00	191	100.00	0.375	183	100.00	7	100.00	190	100.00	0.248
**Age**		156	45.38±2.48	35	49.68±3.97	191	46.17±2.15	0.127	169	45.24±2.34	22	53.32±4.53	191	46.17±2.15	0.018	183	45.81±2.22	7	53.57±7.12	190	46.17±2.15	0.183
**Body Mass Index**		156	27.82±0.76	35	30.08±1.85	191	28.23±0.71	0.015	169	27.97±0.75	22	30.26±2.25	191	28.23±0.71	0.043	183	28.11±0.72	7	30.74±4.93	190	28.21±0.72	0.172
	**NO=1**	78	50.32	24	68.57	102	53.68		88	52.38	14	63.64	102	53.68		96	52.75	5	71.43	101	53.44	
**Smoke**	**EX=2**	31	20.00	3	8.57	34	17.89		33	19.64	1	4.54	34	17.90		34	18.68	0	0.00	34	17.99	
	**YES=3**	46	29.68	8	22.86	54	28.42		47	27.98	7	31.82	54	28.42		52	28.57	2	28.57	54	28.57	
	**Total**	155	100.00	35	100.00	190	100	0.131	168	100.00	22	100.00	190	100.00	0.241		100.00		100.00	189	100.00	0.514
**Pack-years**		153	9.15±2.72	34	9.53±6.13	187	9.22±2.46	0.905	165	8.69±2.60	22	13.2±8.85	187	9.22±2.46	0.245	179	9.28±2.54	7	8.93±14.11	186	9.27±2.47	0.958
	**NO=0**	113	72.44	25	71.43	138	72.25		125	73.96	13	59.09	138	72.25		132	72.13	5	71.43	137	72.11	
**Metabolic comorbidity**	**YES=1**	43	27.56	10	28.57	53	27.75		44	26.04	9	40.91	53	27.75		51	27.87	2	28.57	53	27.89	
	**Total**	156	100.00	35	100	191	100	0.925	169	100.00	22	100.00	191	100.00	0.234	183	100.00	7	100.00	190	100.00	1.000
	**NO=0**	107	68.59	19	54.29	126.2	65.97		115	68.05	11	50.00	126	65.97		121	66.12	4	57.14	125	65.79	
**Cardiovascular comorbidity**	**YES=1**	49	31.41	16	45.71	65	34.03		54	31.95	11	50.00	65	34.03		62	33.88	3	42.86	65	34.21	
	**Total**	156	100.00	35.2	100.00	191.2	100.00	0.176	169	100.00	22	100.00	191	100.00	0.181	183	100.00	7	100.00	190	100.00	0.711
	**NO=0**	125	80.13	24	68.57	149	78.01		134	79.29	15	68.18	149	78.01		144	78.69	4	57.14	148	77.89	
**Respiratory symptoms in childhood**	**YES=1**	31	19.87	11	31.43	42	21.99		35	20.71	7	31.82	42	21.99		39	21.31	3	42.86	42	22.11	
	**Total**	156	100	35	100	191	100	0.174	169	100.00	22	100.00	191	100.00	0.274					190		0.183
	**NO=0**	123	78.85	19	54.29	142	74.35		131	77.51	11	50.00	142	74.35		138	75.41	4	57.14	142	74.74	
**Family history of respiratory diseases**	**YES=1**	33	21.15	16	45.71	49	25.65		38	22.49	11	50.00	49	25.65		45	24.59	3	42.86	48	25.26	
	**Total**	156	100	35	100	191	100	0.005	169	100.00	22	100.00	191	100.00	0.009	183	100.00	7	100.00	190	100.00	0.372
	**NO=0**	78	50.32	12	35.29	90	47.62		81	48.50	9	40.91	90	47.62		88	48.62	2	28.57	90	47.87	
**Wood heating**	**YES=1**	77	49.68	22	64.71	99	52.38		86	51.50	13	59.09	99	52.38		93	51.38	5	71.43	98	52.13	
	**Total**	155	100.00	34	100.00	189	100	0.131	167	100.00	22	100.00	189	100.00	0.651	181	100.00	7	100.00	188	100.00	0.447
	**NO=0**	69	44.23	15	42.86	84	43.98		75	44.38	9	40.91	84	43.98		80	43.72	4	57.14	84	44.21	
**Employed**	**YES=1**	87	55.77	20	57.14	107	56.02		94	55.62	13	59.09	107	56.02		103	56.28	3	42.86	106	55.79	
	**Total**	156	100	35	100	191	100	1.000	169	100.00	22	100.00	191	100.00	0.822	183	100.00	7	100.00	190	100.00	0.702
	**NO=0**	92	58.97	22	62.86	114	59.69		102	60.36	12	54.55	114	59.69		111	60.66	2	28.57	113	59.47	
**Occupational exposure**	**YES=1**	64	41.03	13	37.14	77	40.31		67	39.64	10	45.45	77	40.31		72	39.34	5	71.43	77	40.53	
	**Total**	156	100.00	35	100.00	191	100.00	0.707	169	100.00	22	100.00	191	100.00	0.648	183	100.00	7	100.00	190	100.00	0.122
	**NO=0**	127	81.41	30	85.71	157	82.20		139	82.25	18	81.82	157	82.20		150	81.97	6	85.71	156	82.11	
**Employed in industry**	**YES=1**	29	18.59	5	14.29	34	17.80		30	17.75	4	18.18	34	17.80		33	18.03	1	14.29	34	17.89	
	**Total**	156	100.00	35	100.00	191	100.00	0.633	169	100.00	22	100.00	191	100.00	1.000	183	100.00	7	100.00	190	100.00	1.000
	**Primary (or lower)**	20	13.07	3	8.82	23	12.3		21	12.73	2	9.09	23	12.3		19	12.03	4	13.79	23	12.3	
**Level of education**	**Secondary (or higher)**	133	86.93	31	91.18	164	87.8		144	87.27	20	90.91	164	87.70		139	87.97	25	86.21	164	87.70	
	**Total**	153	100.00		100.00	187	100.10	0.495	165	100.00	22	100.00	187	100.00	0.626	158	100.00	29	100.00	187	100.00	0.790
	**> 500 m**	129	82.69	25	71.43	154	80.63		139	82.25	15	68.18	154	80.63		134	82.72	20	68.97	154	80.63	
**Distance from the main road running through the valley**	**< 500 m**	27	17.31	10	28.57	37	19.37		30	17.75	7	31.82	37	19.37		28	17.28	9	31.03	37	19.37	
	**Total**	156	100.00		100.00	191	100.00	0.128	169	100.00	22	100.00	191	100.00	0.116	162	100.00	29	100.00	191	100.00	0.084

		High-grade dyspnoea (has to stop to take a breath at normal gait on the level							Chronic bronchitis							Bronchial asthma or asthmatic bronchitis						
		NO		YES		TOTAL			NO		YES		TOTAL			NO		YES		TOTAL		
		N	% (or mean)	N	% (or mean)	N	% (or mean)	p	N	% (or mean)	N	% (or mean)	N	% (or mean)	p	N	% (or mean)	N	% (or mean)	N	% (or mean)	p
**Sex**	**M=0**	98	56.00	6	37.50	104	54.45		95	52.78	9	81.82	104	54.45		94	54.34	10	55.56	104	54.45	
	**F=1**	77	44.00	10	62.50	87	45.55		85	47.22	2	18.18	87	45.55		79	45.66	8	44.44	87	45.55	
	**Total**	175	100.00	16	100.00	191	100	0.193	180	100.00	11	100.00	191	100.00	0.069	173	100.00	18	100.00	191	100.00	1.000
**Age**		175	45.27±2.27	16	55.94±4.88	191	46.17±2.15	0.007	180	45.62±2.21	11	55.09±9.01	191	46.17±2.15	0.043	173	46.39±2.28	18	44.00±6.81	191	46.17±2.15	0.523
**Body Mass Index**		175	27.96±0.73	16	31.18±2.92	191	28.23±0.71	0.013	180	28.10±0.73	11	30.48±3.77	191	28.23±0.71	0.125	173	27.94±0.71	18	31.08±3.22	191	28.23±0.71	0.011
**Smoke**	**NO=1**	92	52.87	10	62.50	102	53.68		98	54.75	4	36.36	102	53.68		92	53.49	10	55.55	102	53.68	
	**EX=2**	31	17.82	3	18.75	34	17.90		31	17.32	3	27.28	34	17.89		31	18.02	3	16.67	34	17.90	
	**YES=3**	51	29.31	3	18.75	54	28.42		50	27.93	4	36.36	54	28.42		49	28.49	5	27.78	54	28.42	
	**Total**	174	100.00	16	100.00	190	100.00	0.684	179	100.00	11	100.00	190	100	0.439	172	100.00	18	100.00	190	100.00	1.000
**Pack-years**		172	8.86±2.38	15	13.35±15.54	187	9.22±2.46	0.330	176	8.16±2.25	11	26.18±22.09	187	9.22±2.46	0.001	170	9.26±2.63	17	8.74±7.28	187	9.22±2.46	0.904
**Metabolic comorbidity**	**NO=0**	128	73.14	10	62.50	138	72.25		134	74.44	4	36.36	138	72.25		126	72.83	12	66.67	138	72.25	
	**YES=1**	47	26.86	6	37.50	53	27.75		46	25.56	7	63.64	53	27.75		47	27.17	6	33.33	53	27.75	
	**Total**	175	100.00	16	100.00	191	100.00	0.558	180	100.00	11	100.00	191	100	0.028	173	100.00	18	100.00	191	100.00	0.713
**Cardiovascular comorbidity**	**NO=0**	119	68.00	7	43.75	126	65.97		122	67.78	4	36.36	126	65.97		115	66.47	11	61.11	126	65.97	
	**YES=1**	56	32.00	9	56.25	65	34.03		58	32.22	7	63.64	65	34.03		58	33.53	7	38.89	65	34.03	
	**Total**	175	100.00	16	100.00	191	100.00	0.112	180	100.00	11	100.00	191	100.00	0.097	173	100.00	18	100.00	191	100.00	0.677
**Respiratory symptoms in childhood**	**NO=0**	135	77.14	14	87.50	149	78.01		144	80.00	5	45.45	149	78.01		137	79.19	12	66.67	149	78.01	
	**YES=1**	40	22.86	2	12.50	42	21.99		36	20.00	6	54.55	42	21.99		36	20.81	6	33.33	42	21.99	
	**Total**	175	100.00	16	100.00	191	100.00	0.530	180	100.00	11	100.00	191	100.00	0.016	173	100.00	18	100.00	191	100.00	0.236
**Family history of respiratory diseases**	**NO=0**	131	74.86	11	68.75	142	74.35		136	75.56	6	54.55	142	74.35		131	75.72	11	61.11	142	74.35	
	**YES=1**	44	25.14	5	31.25	49	25.65		44	24.44	5	45.45	49	25.65		42	24.28	7	38.89	49	25.65	
	**Total**	175	100.00	16	100.00	191	100.00	0.561	180	100.00	11	100.00	191	100.00	0.153	173	100.00	18	100.00	191	100.00	0.254
**Wood heating**	**NO=0**	86	49.71	4	25.00	90	47.62		85	47.75	5	45.45	90	47.62		81	47.37	9	50.00	90	47.62	
	**YES=1**	87	50.29	12	75.00	99	52.38		93	52.25	6	54.55	99	52.38		90	52.63	9	50.00	99	52.38	
	**Total**	173	100.00	16	100.00	189	100.00	0.070	178	100.00	11	100.00	189	100.00	1.000	171	100.00	18	100.00	189	100.00	1.000
**Employed**	**NO=0**	80	45.71	4	25.00	84	43.98		79	43.89	5	45.45	84	43.98		77	44.51	7	38.89	84	43.98	
	**YES=1**	95	54.29	12	75.00	107	56.02		101	56.11	6	54.55	107	56.02		96	55.49	11	61.11	107	56.02	
	**Total**	175	100.00	16	100.00	191	100.00	0.123	180	100.00	11	100.00	191	100.00	1.000	173	100.00	18	100.00	191	100.00	0.804
**Occupational exposure**	**NO=0**	106	60.57	8	50.00	114	59.69		108	60.00	6	54.55	114	59.69		107	61.85	7	38.89	114	59.69	
	**YES=1**	69	39.43	8	50.00	77	40.31		72	40.00	5	45.45	77	40.31		66	38.15	11	61.11	77	40.31	
	**Total**	175	100.00	16	100.00	191	100.00	0.435	180	100.00	11	100.00	191	100.00	0.758	173	100.00	18	100.00	191	100.00	0.077
**Employed in industry**	**NO=0**	146	83.43	11	68.75	157	82.20		147	81.67	10	90.91	157	82.20		143	82.66	14	77.78	157	82.20	
	**YES=1**	29	16.57	5	31.25	34	17.80		33	18.33	1	9.09	34	17.80		30	17.34	4	22.22	34	17.80	
	**Total**	175	100.00	16	100.00	191	100.00	0.169	180	100.00	11	100.00	191	100.00	0.692	173	100.00	18	100.00	191	100.00	0.533
**Level of education**	**Primary (or lower)**	20	11.63	3	20	23	12.3		20	11.36	3	27.27	23	12.3		21	12.43	2	11.11	23	12.3	
	**Secondary (or higher)**	152	88.37	12	80	164	87.70		156	88.64	8	72.73	164	87.70		148	87.57	16	88.89	164	87.80	
	**Total**	172	100.00	15	100.00	187	100.00	0.344	176	100.00	11	100.00	187	100.00	0.119	169	100.00	18	100.00	187	100.10	0.872
**Distance from the main road running through the valley**	**> 500 m**	144	82.29	10	62.5	154	80.63		145	80.56	9	81.82	154	80.63		140	80.92	14	77.78	154	80.63	
	**< 500 m**	31	17.71	6	37.5	37	19.37		35	19.44	2	18.18	37	19.37		33	19.08	4	22.22	37	19.37	
	**Total**	175	100.00	16	100.00	191	100.00	0.055	180	100.00	11	100.00	191	100.00	0.918	173	100.00	18	100.00	191	100.00	0.748

		Respiratory allergic symptoms							Respiratory allergic symptoms associated with eye symptoms						
		NO		YES		TOTAL			NO		YES		TOTAL		
		N	% (or mean)	N	% (or mean)	N	% (or mean)	p	N	% (or mean)	N	% (or mean)	N	% (or mean)	p
**Sex**	**M=0**	75	55.97	29	50.88	104	54.45		66	57.39	38	50.00	104	54.45	
	**F=1**	59	44.03	28	49.12	87	45.55		49	42.61	38	50.00	87	45.55	
	**Total**	134	100.00	57	100.00	191	100.00	0.530	115	100.00	76	100.00	191	100.00	0.374
**Age**		134	47.21±2.70	57	43.72±3.44	191	46.17±2.15	0.144	115	46.43±2.88	76	45.76±3.27	191	46.17±2.15	0.764
**Body Mass Index**		134	27.86±0.82	57	29.10±1.42	191	28.23±0.71	0.118	115	27.64±0.88	76	29.14±1.19	191	28.23±0.71	0.042
**Smoke**	**NO=1**	67	50.38	35	61.40	102	53.68		56	49.12	46	60.53	102	53.68	
	**EX=2**	26	19.55	8	14.04	34	17.90		24	21.06	10	13.15	34	17.90	
	**YES=3**	40	30.08	14	24.56	54	28.42		34	29.82	20	26.32	54	28.42	
	**Total**	133	100.01	57	100.00	190	100.00	0.404	114	100.00	76	100.00	190	100.00	0.255
**Pack-years**									112	10.28±3.48	75	7.64±3.33	187	9.22±2.46	0.302
**Metabolic comorbidity**	**NO=0**	-	-	-	-	-	-	-	-	-	-	-	-	-	
	**YES=1**	-	-	-	-	-	-	-	-	-	-	-	-	-	
	**Total**	-	-	-	-	-	-	-	-	-	-	-	-	-	
**Cardiovascular comorbidity**	**NO=0**	-	-	-	-	-	-	-	-	-	-	-	-	-	
	**YES=1**	-	-	-	-	-	-	-	-	-	-	-	-	-	
	**Total**	-	-	-	-	-	-	-	-	-	-	-	-	-	
**Respiratory symptoms in childhood**	**NO=0**	111	82.84	38	66.67	149	78.01		98	85.22	51	67.11	149	78.01	
	**YES=1**	23	17.16	19	33.33	42	21.99		17	14.78	25	32.89	42	21.99	
	**Total**	134	100.00	57	100.00	191	100.00	0.021	115	100.00	76	100.00	191	100.00	0.004
**Family history of respiratory diseases**	**NO=0**	106	79.10	36	63.16	142	74.35		92	80.00	50	65.79	142	74.35	
	**YES=1**	28	20.90	21	36.84	49	25.65		23	20.00	26	34.21	49	25.65	
	**Total**	134	100.00	57	100.00	191	100.00	0.029	115	100.00	76	100.00	191	100.00	0.042
**Wood heating**	**NO=0**	62	46.97	28	49.12	90	47.62		54	47.79	36	47.37	90	47.62	
	**YES=1**	70	53.03	29	50.88	99	52.38		59	52.21	40	52.63	99	52.38	
	**Total**	132	100.00	57	100.00	189	100.00	0.874	113	100.00	76	100.00	189	100.00	1.000
**Employed**	**NO=0**	64	47.76	20	35.09	84	43.98		53	46.09	31	40.79	84	43.98	
	**YES=1**	70	52.24	37	64.91	107	56.02		62	53.91	45	59.21	107	56.02	
	**Total**	134	100.00	57	100.00	191	100.00	0.114	115	100.00	76	100.00	191	100.00	0.552
**Occupational exposure**	**NO=0**	84	62.29	30	52.63	114	59.69		72	62.61	42	55.26	114	59.69	
	**YES=1**	50	37.31	27	47.37	77	40.31		43	37.39	34	44.74	77	40.31	
	**Total**	134	99.60	57	100.00	191	100.00	0.202	115	100.00	76	100.00	191	100.00	0.366
**Employed in industry**	**NO=0**	112	83.58	45	78.95	157	82.20		97	84.35	60	78.95	157	82.20	
	**YES=1**	22	16.42	12	21.05	34	17.80		18	15.65	16	21.05	34	17.80	
	**Total**	134	100.00	57	100.00	191	100.00	0.535	115	100.00	76	100.00	191	100.00	0.342
**Level of education**	**Primary (or lower)**	19	14.5	4	7.14	23	12.3		15	13.27	8	10.81	23	12.3	
	**Secondary (or higher)**	112	85.50	52	92.86	164	87.70		98	86.73	66	89.19	164	87.70	
	**Total**	131	100.00	56	100.00	187	100.00	0.16	113	100.00	74	100.00	187	100.00	0.616
**Distance from the main road running through the valley**	**> 500 m**	107	79.85	47	82.46	154	80.63		94	81.74	60	78.95	154	80.63	
	**< 500 m**	27	20.15	10	17.54	37	19.37		21	18.26	16	21.05	37	19.37	
	**Total**	134	100.00	57	100.00	191	100.00	0.677	115	100.00	76	100.00	191	100.00	0.633

Legend: M: Males; F: Females; N: Number.

© 2018 by the authors. Submitted for possible open access publication under the terms and conditions of the Creative Commons Attribution (CC BY) license (http://creativecommons.org/licenses/by/4.0/).
